# Does the Clinical Form of *Oral Lichen Planus* (OLP) Influence the Oral Health–Related Quality of Life (OHRQoL)?

**DOI:** 10.3390/ijerph17186633

**Published:** 2020-09-11

**Authors:** Linda Daume, Constance Kreis, Lauren Bohner, Johannes Kleinheinz, Susanne Jung

**Affiliations:** Department of Cranio-Maxillofacial Surgery, University Hospital Münster, Albert-Schweitzer-Campus 1, Building W 30, D-48149 Münster, Germany; constance.kreis@ukmuenster.de (C.K.); lauren.bohner@ukmuenster.de (L.B.); johannes.kleinheinz@ukmuenster.de (J.K.); susanne.jung@ukmuenster.de (S.J.)

**Keywords:** *oral lichen planus*, quality of life, OHIP-14, oral health

## Abstract

(1) Background: The aim of the study was to investigate the oral health–related quality of life (OHRQoL) of patients with *oral lichen planus* (OLP) and to evaluate differences between the various clinical forms of OLP. Specifically, the differences in OHRQoL, physical pain levels and eating restriction were assessed; (2) Methods: One hundred and twelve patients with clinical and histological features of OLP from the Department of Cranio-Maxillofacial Surgery of the Münster University Hospital participated in this prospective study. OHRQoL was analysed by using the German short version of the Oral Health Impact Profile (OHIP-14). Physical pain levels and restriction in eating were rated on visual analogue scales (VAS). The statistical analysis was performed using the Mann–Whitney U-Test and the chi-squared test with a significance level at *p* = 0.05; (3) Results: Group 1 consisted of patients with reticular OLP (*n* = 50) and group 2 of patients with atrophic, erosive-ulcerative or bullous OLP (*n* = 62). The average OHIP-14 score was 13.54 points and differed significantly between the two groups. There were significant differences in the domains “physical pain”, “psychological discomfort”, “physical disability” and “social disability”. The VAS “physical pain” score and “restriction in eating” score varied significantly between the clinical forms. Positive correlations were found between the OHIP-14 total scores and the VAS scores; (4) Conclusion: The OHRQoL is significantly limited in patients with OLP; especially, patients with erosive-ulcerative OLP are affected.

## 1. Introduction

*Oral lichen planus* (OLP)is a chronic inflammatory autoimmune oral mucosa disease. A recently published review shows a worldwide prevalence of 1.01% with being more prevalent in women than in men [1,2]. The risk of malignant transformation is 1.2% [3]. The etiology is unclear but a T-cell-mediated chronic inflammatory oral mucosal disease is discussed. Various mechanisms as antigen-specific and non-specific are hypothesised to be involved in the pathogenesis [4].

The clinical picture of OLP varies from small barely visible fine white lesions to large areas that can affect the entire oral mucosa. The symptoms are variable, ranging from no symptoms to severe intraoral pain. About two-thirds of the patients describe a burning sensation and pain in the area of the oral mucosa, which leads to difficulties and restrictions in eating, speaking and swallowing [4]. Natural courses of OLP usually involve active disease phases and periods of remission. According to Andreasen’s clinical classification, reticular, papular, plaque, atrophic, erosive-ulcerative and bullous forms can be distinguished [5].

The therapy of OLP has no curative approach. The aim is to reduce the symptomatic lesions and to alleviate the symptoms [2].

So far hardly any studies have investigated the effects upon quality of life of affected patients. Since it is a chronic disease with recurrent symptoms and lesions, many of the affected patients not only have significant oral limitations, but have social and psychological impairments [6].

Oral health–related quality of life (OHRQoL) is a useful tool for measuring the impact of oral diseases and associated treatments based on patients’ own perception. This subjective perception is important for the assessment of treatment needs, clinical situation and therapy planning [7,8].

Previous studies examined OHRQoL in OLP patients and found significant impairment [6,7,9,10]. A clear negative impact on quality of life was recorded by Parlatescu et al. [11]. In particular, patients with erosive and bullous OLP had a significantly lower OHRQoL than those with reticular OLP [7].

The aim of this study is to establish correlations between OHRQoL and the clinical forms of OLP. The total and subscale scores of the short form of the German version of the Oral Health Impact Profile (OHIP-14) are examined. The extent to which physical oral mucosal pain and restriction in eating affect OHRQoL in patients with OLP is analysed.

## 2. Materials and Methods

Patients with OLP who visited the Department of Cranio-Maxillofacial Surgery of the Münster University Hospital for periodic clinical control in 2019 were included in this prospective observational study. Inclusion criteria was a clinically and histopathologically proven OLP based upon modified WHO diagnostic criteria [12].

Besides that a minimum age of 18 years and a good knowledge of spoken and written German were necessary. Patients with other confirmed oral mucosal diseases and oral lichenoid lesions and patients after radiation were excluded. At the time of investigation, patients did not receive topical or general treatment but all patients indicated acute symptoms.

The study was approved by the Ethics Committee of the Medical Association of Westphalia-Lippe and the Westphalian Wilhelms University Münster (Ref. No. 2019-033.f-S). After a detailed explanation of the purpose of this study given by the doctors, verbal and written consent was obtained from each patient. The patients were clinically examined and the findings were documented through writing and photodocumentation. The clinical examination and diagnosis were carried out under standardised conditions by an oral surgeon.

**Figure 1:**
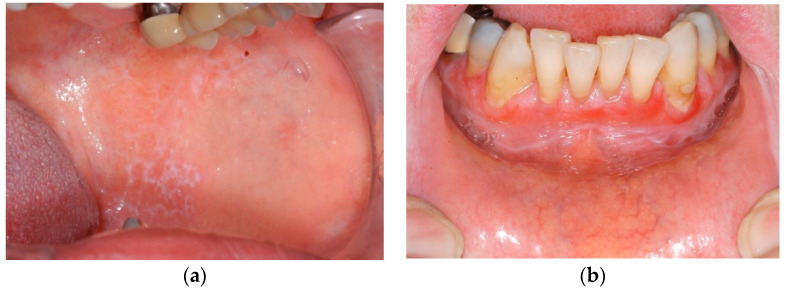
Clinical patterns of *oral lichen planus* (OLP): (**a**) reticular and (**b**) erosive-ulcerative.

The two clinical groups in this study are based on Andreasen’s clinical classification. Group 1 represents the white variants of OLP (reticular, papular and plaque-type). They are known as the “hypokeratotic” forms (Figure 1a). The red variants of OLP are the erosive-ulcerative, the bullous and the atrophic forms. They are the so called “erosive” forms and are summarised in group 2 (Figure 1b). The most frequent clinical variant was the reticular form (group 1). Papular and plaque-type OLP did not occur in our group of patients.

Patients received a questionnaire with open questions and the German version of the OHIP-14 questionnaire (see Appendix A Table A1) to evaluate subjective OHRQoL [13]. Additionally, anamnestic data on age and gender were collected. The questionnaires were completed and evaluated anonymously. The OHIP-14 questionnaire contains 14 items divided into 7 domains of questions (“functional limitation”, “physical pain”, “psychological discomfort”, “physical disability”, “psychological disability”, “social disability” and “handicap”). The possible answers concerning reduced quality of life are given on a “likert-type” scale (0 = never, 1 = hardly ever, 2 = occasionally, 3 = often and 4 = very often). A maximum of 56 points can be obtained, 8 points in each subgroup. The higher the overall score, the worse the OHRQoL. The OHIP-14 summary score is therefore a problem index.

Compared to the study of Parlatescu et al., the answer options “very often” and “often” were combined and analysed in order to evaluate the negative impact [11].

In addition, patients rated current physical pain and restriction in eating on a visual analogue scale (VAS) [14]. Restriction in eating referred particularly to the consumption of spicy or hard food. On the visual analogue scale, the patient had to rate his/her sensation on a scale from 0 to 10. The scale was given as a bar of 10 cm on which the patient marked the intensity of sensation as a distance from the left edge (0 cm = no pain/difficulty, 10 cm = most pain/difficulty).

### Statistical Analysis

Statistical analysis was performed by using SPSS Software 26.0 (IBM-Group, Armonk, NY, USA: IBM Corp.). The OHIP-14 results were compared to the Mann–Whitney U-Test because the data was non-normally distributed. Categorical data was analysed by using the chi-squared test. The exact test according to Fisher was used for expected frequencies of less than 5. Correlations between the OHIP-14 score and the VAS scores were investigated using the Spearman’s rho correlation coefficient. The following grading of the degree of correlation was applied: 0.2–0.5 marks a low, 0.5–0.7 a moderate, 0.7–0.9 a good and 0.9–1.0 a high correlation. The statistical significance level was set at *p* < 0.05.

## 3. Results

A total of 112 patients (21 male, 91 female) with confirmed OLP participated in this study. Significantly more women were examined in both groups (*p* < 0.01). The mean age was 59.98 ± 10.69 (26.6–85.3) years.

The reticular form occurred in 44.64% of all cases (*n* = 50, group 1). The erosive-ulcerative form, the bullous form and the atrophic form were present in 55.36% of all cases (*n* = 62, group 2).

The chi-squared test showed a significant age difference between the two groups (*p* = 0.001). Patients with the reticular form of OLP were younger. The demographic data are summarised in Table 1.

The average total OHIP-14 value was 13.54 (±10.91) points. Patients with reticular OLP had significantly lower OHIP-14 values than patients with other forms (*p* = 0.02) (Table 2).

Regarding the subscale OHIP-14 scores, differences in the domains of “physical pain”, “psychological discomfort”, “psychological disability” and “social disability” were statistically significant between reticular and non-reticular forms (Table 2). The highest scores were found for the items “painful aching” and “uncomfortable to eat”.

The overall average VAS physical pain score was 4.31 ± 2.63 points. Patients in group 1 had a significantly lower score than patients in group 2 (*p* < 0.01). The VAS restriction in eating score was also significantly higher in patients with non-reticular OLP (*p* = 0.02) (Table 2).

A positive Spearman rho correlation was found between the total OHIP-14 scores and the VAS pain scores in both OLP groups. When comparing the VAS restriction in eating scores with the OHIP-14 scores, there was a good positive correlation in the overall comparison and in group 1 (reticular OLP). In group 2, there was a moderate positive Spearman rho correlation (Table 3).

The number of negative impact reported on OHIP-14 items was analysed (Table 4). The highest percentage values were achieved in the category “physical pain”, “psychological discomfort” and “psychological disability”. There were no significant differences between group 1 and 2.

## 4. Discussion

The aim of this study was to investigate the level of OHRQoL in a cohort of 112 biopsy-proven OLP as indicated by OHIP-14. The difference in OHRQoL between patients with reticular OLP and non-reticular OLP was assessed. Further physical pain and restriction in eating were studied using VAS.

Our results showed that patients with OLP, in general, have a significantly reduced OHRQoL. The clinical form of OLP significantly influences OHRQoL. The quality of life of patients with reticular lichen planus was significantly less affected than the comparator group.

The reticular form is the most common form of OLP and is also known as “mild” form. The other forms (group 2) were categorised as “severe” forms [15]. Long-term studies assume that the clinical form changes over time. The reticular lesions may transform into the erosive-ulcerative lesion over time [16]. Further information on transformation and age need to be verified in future long-term studies with larger study populations.

The age and gender distribution showed that middle-aged women are particularly affected by OLP in general. This has also been demonstrated in 2019 in a number of other studies [17,18]. The cause of the higher prevalence in female patients has not been conclusively clarified yet.

### 4.1. OHIP-14

In order to assess our OHRQoL results, they have to be compared with reference values of the general population. John et al. determined an average OHIP-14 value of 4.09 points for the general population in Germany [19].

Patients with OLP achieved an average OHIP-14 value of 13.54 points. In the examined group 2 (erosive-ulcerous, bullous, atrophic OLP), the value was even higher with 15.55 points. This shows the significant reduction of quality of life of patients with OLP. This influence on quality of life, especially associated with erosive-ulcerous and bullous forms, was proven in various studies. Often comparative studies between OLP and other oral diseases were examined [6,9].

So far, there are no defined reference values for distinguishing healthy and diseased patients. With the OHIP questionnaire, Reissmann et al. and Locker et al. examined the benefits of general prosthetic and dental therapies. They found out that after dental therapy the minimum perceptible OHIP score change was 2 points and 4.3 points [20,21]. This means it is unclear at which point the OHIP score change is clinically relevant and might state a pathological aspect in a patient’s life.

Furthermore, additional studies show that the OHIP 14 values of patients with OLP fluctuate between 9.42 and 21.7 [9,22]. Differences are due to the methodology, especially concerning the differences in study population or study design.

A comparable study by Parlatescu et al. achieved 11.77 points in patients with reticular OLP and 15.68 points in atrophic, erosive, ulcerative and bullous OLP forms [11]. These severe forms cause pain in the oral cavity and lead to discomfort and restrictions in eating, speaking, swallowing and oral care.

Aksoy and colleagues also achieved elevated OHIP-14 values (8.5 points on average). The median OHIP-14 value in patients with reticular OLP was 8 points and in patients with non-reticular OLP 12 points [23].

Lundquist et al. examined patients with oral and genital erosive lichen planus and found a high degree of psychological impairment (depression, anxiety and stress). When differentiating the clinical lichen forms, patients with reticular OLP were significantly less affected [24].

Psychological aspects influence the course of OLP. Anxiety and depression are common cofactors in OLP [25]. In a study by Wiriyakijja et al., prevalences of anxiety, depression and distress were 39.23%, 20.77% and 27.69%, respectively [26].

The long-term, chronic and unpredictable course of the disease has a strong impact on the psyche of patients. Moreover, the potential risk of malignant transformation, which is associated with OLP, has an extra negative effect. Ni Riordain et al. investigated the influence of chronic oral mucosal diseases on the daily life of patients. The effects have been described in a variety of areas, from physical health and functioning to concern for the future [27].

### 4.2. OHIP-14 Domains

Looking at the 7 domains of the OHIP-14, the category of “physical pain” was most frequently affected (3.58 ± 2.42 points). The highest scores were obtained for individual questions about “painful aching” and “uncomfortable to eat” (Table 2). “Physical pain” and “psychological discomfort” were most clearly affected in the studies by Parlatescu et al. The study by Karbach et al. showed similar results [9,11]. Here, “physical pain” and “physical disability” were the most affected. Likewise, in the study by Aksoy and colleagues, the highest values were achieved in the categories “physical pain” and “psychological disability” [23].

The differences in the categories “physical pain”, “psychological discomfort”, “psychological disability” and “social disability” were significant between the clinical forms (groups 1 and 2). Similar results were given by Parlatescu and colleagues who achieved significant differences in almost all subcategories [11].

### 4.3. VAS Scores

The VAS scores differed significantly between the groups in favor of reticular lichen forms.

Group 1 and 2 achieved a moderate positive Spearman correlation between VAS pain values and OHIP values. Numerous other studies support this correlation between quality of life and pain. The more severe the pain, the greater the limitations on quality of life [6,9,11]. Zuo and colleagues even found a highly significant correlation [22].

VAS restriction in eating scores showed a medium (group 2) or high (group 1) correlation with OHIP-14 (Table 4). Complaints about food intake make healthy eating more difficult and also affect the patients’ social lives resulting in a reduced quality of life and psychological impairment. A study of the self-assessment of oral health, satisfaction with overall health and quality of life of patients with OLP compared to a healthy control group found that those affected suffered particularly from the negative effects of daily life and restrictions of eating [28].

### 4.4. Negative Impact on OHIP-14

Comparing only the negative aspects of the questionnaire, the categories of “physical pain”, “psychological discomfort” and “psychological disability” were mentioned as the most severe.

Signs of anxiety, depression and distress were examined in a study by Wiriyakijja et al. One-third of 260 patients named psychological complaints. Patients with these mental concomitant diseases had significantly more frequent physical pain in the mouth area than patients without concomitant diseases [26]. As already known for other chronic diseases, somatic complaints have an impact on the psyche [29].

The frequency of the occurrence of negative impacts in patients with oral mucosal diseases was investigated by Liu et al. Patients with oral mucosal diseases had significantly higher numbers of negative impacts on OHIP-14 in comparison with healthy patients. However, the study did not specifically examine patients with OLP [6]. The clinical forms of OLP were analysed in the study by Parlatescu et al. with regard to the negative impacts. They found significant differences in almost all OHIP-categories. The consequences for OHRQoL were significantly lower in patients with reticular OLP [11]. This is consistent with our results, but the differences were not significant (Table 4).

The evaluation of OHRQoL using the OHIP-14 and the VAS scale is simple, fast and reliable. The OHIP-14 has been used in numerous studies to assess the quality of life in patients with OLP [6,7,8,11]. The VAS scale is also simple, easy to understand and has a good compliance [14].

The quality of life is increasingly used as a valid, appropriate and significant indicator for public health research and practice. Health-related quality-of-life measures, including objective and subjective assessments, are particularly useful for evaluating the effectiveness of therapies for chronic diseases. The OHIP-14 questionnaire is a questionnaire for measuring subjective oral health–related dysfunction, discomfort and disability and is based on a conceptual oral health model developed by Locker et al. [21].

The patient’s own perception of quality of life could be included in the clinical treatment process. This would increase the clinician’s awareness of how the disease affects the patient on a daily basis. Clinical measures alone do not provide an accurate picture of the impact of oral health on an individual’s quality of life. Similarly, the treatment of clinical factors alone does not always provide effective symptom relief. The aim must be to consider all aspects of health [30]. According to the literature, oral health problems can impact various aspects of life, such as causing pain, discomfort and eating problems. Furthermore, individuals with more depressive symptoms report a worse oral quality of life than mentally healthy patients. As a consequence, this affects interpersonal relationships negatively and it may shape an individual’s behaviour and negative self-portrait [31].

#### Limitations

First, our sample included only patients from one dental clinic, which limits generalisation. Further, our study used only one OHRQoL index and the fact that the change in OHRQoL following clinical response to treatment was not determined in our study.

In the future, this will have to be verified on the basis of larger case numbers for evidence-based conclusions and needs to be compared with a healthy control group. In addition, therapeutic effects should also be assessed by using OHRQoL.

## 5. Conclusions

The oral health–related quality of life of patients with OLP is clearly compromised. This leads to physical, social and psychological consequences that affect everyday life, especially social interactions. However, the clinical form of OLP is an important indicator for assessing the impact of the disease on quality of life. Patients with reticular OLP have a higher quality of life than other clinical forms.

The relevance of OHRQoL is demonstrated and should be included in therapy. The use of oral health–related quality measurements can be an additional tool for screening possible comorbidities. The patient and his or her well-being must be considered as a whole and cannot only be evaluated by using clinically visible parameters.

## Figures and Tables

**Table 1:** Demographic data of study participants with OLP according to the clinical form of disease.

Variables	Total (*n* = 112)	Group 1 (*n* = 50)	Group 2 (*n* = 62)	*p*-Value *
**Age (%)**				
18–60	52 (46,42)	33 (66)	19 (30,65)	<0.01 *
>60	60 (53.57)	17 (34)	43 (69.35)	
**Gender (%)**				
Female N	91 (81.25)	38 (76)	53 (85.48)	0.201
Males N	21 (18.75)	12 (24)	9 (14.52)	

* indicates statistical significance at *p* < 0.05 (chi^2^ test).

**Table 2:** Analysis of responses to Oral Health Impact Profile (OHIP-14) domains and VAS according to the clinical form of disease.

Variables	Total (*n* = 112)		Group 1 (*n* = 50)		Group 2 (*n* = 62)		*p*-Value *
	Median IQR	Mean (±SD)	Median IQR	Mean (±SD)	Median IQR	Mean (±SD)	
OHIP-14							
Functional limitation	1.22 ± 1.58	1 (0–7)	1.12 ± 1.56	0.5 (0–5)	1.31 ± 1.61	1 (0–7)	0.44
*Trouble pronouncing*	0.38 ± 0.84	0 (0–4)	0.36 ± 0.85	0 (0–3)	0.4 ± 0.39	0 (0–4)	0.52
*Taste worsened*	0.84 ± 1.05	0 (0–4)	0.76 ± 1.02	0 (0–4)	0.9 ± 1.07	0 (0–4)	0.46
Physical pain	3.58 ± 2.42	4 (0–8)	2.68 ± 2.19	3 (0–8)	4.31 ± 2.37	5 (0–8)	<0.01 *
*Painful aching*	1.83 ± 1.31	2 (0–4)	1.32 ± 1.29	1 (0–4)	2.24 ± 1.2	2 (0–4)	<0.01 *
*Uncomfortable to eat*	1.75 ± 1.35	2 (0–4)	1.36 ± 1.23	1 (0–4)	2.06 ± 1.37	2 (0–4)	<0.01 *
Psychological discomfort	2.42 ± 2.08	2 (0–8)	2.04 ± 2.14	2 (0–7)	2.73 ± 2.0	3 (0–8)	0.04 *
*Self-conscious*	1.23 ±1.17	1 (0–4)	1.0 ± 1.18	1 (0–4)	1.42 ± 1.14	1 (0–4)	0.04 *
*Tense*	1.19 ±1.16	1 (0–4)	1.04 ± 1.18	1 (0–4)	1.31 ± 1.14	1 (0–4)	0.17
Physical disability	1.61 ± 1.79	1 (0–7)	1.08 ± 1.53	0 (0–7)	2.03 ± 1.87	2 (0–7)	<0.01 *
*Diet unsatisfactory*	0.96 ± 1.09	1 (0–4)	0.64 ± 0.89	0 (0–3)	1.21 ± 1.18	1 (0–4)	<0.01 *
*Interrupt meals*	0.65 ± 0.94	0 (0–4)	0.44 ± 0.84	0 (0–4)	0.82 ± 0.98	1 (0–4)	0.01 *
Psychological disability	1.88 ± 1.80	1 (0–6)	1.78 ± 1.89	1 (0–6)	1.97 ± 1.75	1.5 (0–6)	0.35
*Difficult to relax*	1.29 ± 1.24	1 (0–4)	1.24 ± 1.32	1 (0–4)	1.34 ± 1.19	1 (0–4)	0.55
*Embarrassed*	0.59 ± 0.91	0 (0–3)	0.54 ± 0.86	0 (0–3)	0.63 ± 0.95	0 (0–3)	0.62
Social disability	1.46 ± 1.81	0.5 (0–7)	1.18 ± 1.76	0 (0–6)	1.69 ± 1.83	1.5 (0–7)	0.08
*Irritable*	0.85 ± 1.02	0 (0–4)	0.62 ± 0.95	0 (0–3)	1.03 ± 1.06	1 (0–4)	0.03 *
*Difficulty doing jobs*	0.62 ± 0.9	0 (0–3)	0.56 ± 0.91	0 (0–3)	0.66 ± 0.9	0 (0–3)	0.4
Handicap	1.37 ± 1.59	1 (0–8)	1.18 ± 1.7	0 (0–6)	1.52 ± 1.49	1 (0–8)	0.07
*Life less satisfying*	1.05 ± 1.11	1 (0–4)	0.84 ± 1.13	0 (0–4)	1.23 ± 1.08	1 (0–4)	0.55
*Unable to function*	0.31 ± 0.72	0 (0–4)	0.29 ± 0.77	0 (0–3)	0.34 ± 0.69	0 (0–4)	0.73
Total	13.54 ± 10.91	11 (0–45)	11.06 ± 10.88	16 (0–45)	15.55 ± 10.6	7 (0–43)	0.02 *
VAS-Score							
*Physical pain*	4.31 ± 2.63	4 (0–10)	3.5 ± 2.57	4 (0–10)	4.31 ± 2.52	5 (0–10)	<0.01 *
*Restriction in eating*	3.47 ± 2.86	3 (0–10)	2.72 ± 2.37	2 (0–8)	4.08 ± 3.09	4 (0–10)	0.02 *

* indicates statistical significance at *p* < 0.05 (Mann–Whitney U-Test).

**Table 3:** Comparison of the correlations according to the clinical form of OLP.

Variables	Total (*n* = 112)		Group 1 (*n* = 50)		Group 2 (*n* = 62)	
	r ^†^	*p*-Value *	r ^†^	*p*-Value *	r ^†^	*p*-Value *
OHIP-14-VAS-physical pain	0.63	0.01 *	0.61	<0.01 *	0.60	<0.01 *
OHIP-14-VAS-restriction in eating	0.72	<0.01 *	0.73	<0.01 *	0.67	<0.01 *

* indicates statistical significance at *p* < 0.05 (Mann–Whitney U-Test); ^†^ Spearman correlation coefficients.

**Table 4:** Number of subjects reporting negative social impact by OHIP-14.

Variables	Total (*n* = 112)	Group 1 (*n* = 50)	Group 2 (*n* = 62)	*p*-Value *	
OHIP-14	Number (%)				
Functional limitation	6 (5.36)	3 (15)	3 (4.84)	1.0	^a^
Physical pain	43 (38.39)	11 (22)	32 (51.61)	0.62	^b^
Psychological discomfort	20 (17.86)	7 (14)	13 (20.97)	0.09	^b^
Physical disability	9 (8.04)	1 (2)	8 (12.9)	0.14	^b^
Psychological disablitiy	13 (11.61)	6 (12)	7 (11.29)	0.59	^a^
Social disability	8 (7.14)	3 (6)	5 (8.06)	0.64	^b^
Handicap	6 (5.36)	2 (4)	4 (6.45)	0.22	^b^

* indicates statistical significance at *p* < 0.05, ^a^ chi-squared-test, ^b^ Fisher test.

## Appendix A

**Table A1:** OHIP-14 questionnaire

In the Last Month
Have you had trouble pronouncing any words because of problems with your teeth, mouth or dentures?
Have you felt your sense of taste has worsened because of problems with your teeth, mouth or dentures?
Have you had painful aching in your mouth?
Have you found it uncomfortable to eat any foods because of problems with your teeth, mouth or dentures?
Have you been self-conscious because of your teeth, mouth or dentures?
Have you felt tense because of problems with your teeth, mouth or dentures?
Has your diet been unsatisfactory because of problems with your teeth, mouth or dentures?
Have you had to interrupt meals because of problems with your teeth, mouth or dentures?
Have you found it difficult to relax because of problems with your teeth, mouth or dentures?
Have you been a bit irritable with other people because of problems with your teeth, mouth or dentures?
Have you had difficulty doing your usual jobs because of problems with your teeth, mouth or dentures?
Have you felt that life in general was less satisfying because of problems with your teeth, mouth or dentures?
Have you been totally unable to function because of problems with your teeth, mouth or dentures?
